# Plant Polygalacturonases Involved in Cell Elongation and Separation—The Same but Different?

**DOI:** 10.3390/plants3040613

**Published:** 2014-12-09

**Authors:** Yashodar Babu, Martin Bayer

**Affiliations:** Department of Cell Biology, Max Planck Institute for Developmental Biology, Spemannstrasse 35, Tuebingen 72076, Germany; E-Mail: yashodar.babu@tuebingen.mpg.de

**Keywords:** polygalacturonase, pectin, cell wall, cell elongation, genetic redundancy

## Abstract

Plant cells are surrounded by the primary cell wall, a rigid framework that needs to be modified in order to allow cell growth. Recent data suggest that in addition to the cellulose-hemicellulose network, the pectin matrix plays a critical role in determining the elasticity of the primary cell wall. Polygalacturonases are key homogalacturonan-hydrolyzing enzymes that function in a wide range of developmental processes. In this review, we present recent progress in understanding the role of polygalacturonases during cell elongation and separation. In discussing the specificities and possible redundancies of polygalacturonases, we focus particularly on newly discovered Arabidopsis mutants that have measurable loss-of-function phenotypes. However, data from other species are included when necessary.

## 1. Introduction

In plants, cells are surrounded by a rigid cell wall and are, therefore, fixed in their relative position. As a consequence, the overall shape of the plant body is created by a tightly controlled interplay of cell division and anisotropic cell expansion. The driving force for cell expansion is turgor pressure and it requires controlled relaxation of the cell wall while maintaining cellular integrity [1]. The high osmotic pressure inside the cell, ranging from 0.3 to 1.2 MPa in the case of *Arabidopsis thaliana*, makes this a non-trivial task [2]. Secondary cell-wall material can fortify cell walls after cell elongation has ceased but it is the primary cell wall of meristematic and elongating cells that needs to be modified to control the shape of the plant body [3].

The typical primary cell wall is a complex matrix composed of polysaccharides, a variety of proteins, and phenolics with cellulose, hemicelluloses, and pectin almost equally representing the main polysaccharide content [4]. A major constituent of this composite network is cellulose, a linear β-1,4-glucan, which co-crystalizes with other cellulose polymers into microfibrils, long, rigid fibers that can wind around the cell many times [1,5]. In seed plants (spermatophytes), the most abundant hemicelluloses in primary cell walls are usually xyloglucans (XG). However, the commelinid monocots, which include the grass family, are an exception, as in this group glucuronorarabinoxylans generally predominate [6]. Hemicelluloses are thought to cross-link cellulose microfibrils, thereby creating a rigid network while at the same time enforcing certain spacing between microfibrils to adjust primary cell wall flexibility [1,6].

In this view, the cellulose-xyloglucan network is the main structural component with load-bearing properties while pectin acts as filler matrix similar to composite materials of fiber-reinforced polymers. The extensibility of this network is modified by wall-loosening expansins, which are thought to act on the non-covalent interactions between cellulose microfibrils, as well as XG *endo*-transglycosylases [1].

This traditional model has recently been challenged by data that suggest that only a minor portion of XG is bound directly to cellulose [7] and that pectin displays load-bearing properties in the absence of XG. Therefore the pectin matrix seems to play a more important role in determining the extensibility of the primary cell wall than previously recognized [7,8,9].

Pectin consists of complex high molecular weight polysaccharides that can form hydrated gels. It can be classified into domains of homogalacturonan (HG), rhamnogalacturonan I (RG I) and RG II, and xylogalacturonan depending on the backbone and the degree of branching. The most abundant pectin form is homogalacturonan, a linear polymer of α-1,4-linked D-galacturonic acid [10]. It is synthesized in the *cis*-Golgi by galacturonosyltransferases (GAUTs) and usually highly methyl-esterified by pectin methyltransferases (PMTs) and to lesser degree acetylated by pectin acetyltransferases (PATs) [11]. The degree of methylesterification and acetylation plays a critical role in determining the stiffness of the cell wall since negative charges on the HG backbone can cross-link HG polymers via calcium ions [12]. Pectin methylesterases (PMEs) can hydrolyze methylester bonds and therefore have the potential to increase the degree of HG cross-linking. Interestingly, it was shown that ectopic, ubiquitous expression of PME inhibitors leads to stiffening of cell walls while ectopic expression of PMEs leads to softening [13,14]. This might be explained by competition between different HG sites for binding of calcium ions where newly de-esterified sites lead to the disruption of existing, load-bearing cross-links [9]. On the other hand, the de-esterification might also allow hydrolyzing enzymes like polygalacturonases (PGs) to act on the HG chain leading to softening of the cell wall [12]. Since the amount, structure and modification of pectin influences the physical properties of the cell wall, growth control most likely requires fine-tuning of all these variables. A more detailed description of pectin biosynthesis and structure as well as its modifications during cell growth can be found in the following comprehensive reviews [11,12,15,16,17].

## 2. Polygalacturonases

Polygalacturonases belong to the glycosyl hydrolase family 28 and are key HG hydrolyzing enzymes that have been implicated with a wide range of plant developmental processes such as cell elongation, organ abscission, fruit ripening, microspore release, and pollen tube growth [18]. Plant PG genes belong to large gene families and their expansion and diversification can be attributed to whole genome and segmental duplications in association with gene loss, as well as intron gain and (more predominantly) intron loss events [19,20]. Phylogenetic analysis of PG gene structure in Arabidopsis, rice, and other plant species reveals five distinct clades of PG genes which can be further divided into subclades indicating the occurrence of at least four ancestral PG genes before the divergence of monocots and dicots. While sequences from different clades are relatively divergent, they are rather conserved within a clade and tandem-duplicated genes generally fall into the same subclade [19,20,21,22]. Depending on their mode of action, *endo*- and *exo*-polygalacturonases can be distinguished [23]. *Endo*-PGs hydrolyze the HG polymer at random sites but require at least four consecutive GalA residues of the HG chain to be de-methylesterified [24,25]. Thus, the methylation pattern of the HG chains directly influences possible *endo*-PG-mediated HG cleavage. *Endo*-PG activity might lead to complete hydrolysis of pectin polymers and has therefore the potential to cause rapid cell elongation or even cell separation [12,26].

*Exo*-PGs on the other hand attack the free ends of de-methylesterified HG polymers and thereby reduce the overall polymer length. It has been speculated that the resulting modification of the pectin matrix might be subtler than the random cleavage by *endo*-PGs and might, therefore, be used to fine-tune the extensibility of the primary cell wall [27].

## 3. Polygalacturonases Involved in Fruit Ripening and Cell Separation

Early on, polygalacturonases were isolated from ripening fruits which implied a role in pectin degradation for tissue softening [28]. In a pioneer work in tomato, down-regulation of polygalacturonase expression by an anti-sense construct lead to decreased de-polymerization of solubilized pectins and increased storage-life of ripe fruits although there was no measureable effect on fruit softening [29,30]. Silencing of PG expression in apple and strawberry on the other hand increased the firmness of the ripe fruit significantly but did not change other ripening parameters. It could be shown that in these transgenic plants, ionically and covalently bound pectin exhibits a lower degree of de-polymerization. Microscopic analysis of transgenic fruits revealed smaller intra-cellular spaces and more cellular adhesion [31,32,33]. This is in agreement with earlier reports demonstrating that pectin degradation by PGs also plays a central role in cell separation in abscission events and dehiscence zones [34,35,36]. In apples, constitutive expression of the fruit-specific MdPG1 gene resulted in a range of novel developmental phenotypes including premature leaf shedding due to reduced cell adhesion in abscission zones, malformed leaves and malfunctioning stomata. As a consequence of the strong constitutive expression of MdPG1 in transgenic apple trees, a decrease of the average molecular weight of pectin chains could be demonstrated [37]. In rice, over-expression of *OsBURP16*, the non-catalytic PG1β-subunit of the polygalacturonase PG1, reduced cell adhesion in leaves and pectin content [38]. Together these data support the involvement of PGs in cell separation events in plants.

Many polygalacturonase genes are highly expressed in reproductive tissue [21], and for some, their involvement in cell separation events could be demonstrated by loss-of-function phenotypes: The *endo*-PG QUARTET3 (QRT3) functions in degrading the pollen mother cell wall during microsporogenesis and thus enables the release of unicellular microspores [39]. ARABIDOPSIS DEHISCENCE ZONE POLYGALACTURONASE1 (ADPG1), ADPG2, and QRT2 were reported to act in a redundant manner in anther dehiscence while ADPG2 and QRT2 function partially redundant in floral organ abscission [40].

## 4. Polygalacturonases Involved in Cell Expansion

The previously mentioned examples of polygalacturonases involved in fruit ripening and cell separation might lead to the conclusion that the primary role of *endo*-PGs is the more or less complete breakdown of the pectin matrix in terminal developmental situations like tissue softening, abscission, or dehiscence. This is clearly not the whole story, since, recently, the *endo*-PG POLYGALACTURONASE INVOLVED IN EXPANSION1 (PGX1) was shown to be involved in hypocotyl elongation and floral patterning [41]. Furthermore, *in vivo* assays for pectin-degrading enzymes suggested that during cotyledon expansion in cotton *endo*-PG and *exo*-PG activity could both be detected. Interestingly, their appearance differed temporally during cell elongation with high *endo*-PG activity at an early phase, followed by an increase of *exo*-PG activity during a later phase when *endo*-PG activity decreased [26]. This implies a scenario where different pectin hydrolyzing enzymes might act on the same substrate but in a consecutive manner.

The pectinase LeXPG1 was isolated from tomato seed protein extracts and gene expression was detected in the embryonic root, the developing vasculature as well as the embryo-surrounding endosperm. Based on this expression pattern, it was suggested that LeXPG1 might play a role in cell elongation as well as tissue softening in the embryo and the endosperm, respectively. Interestingly, LeXPG1 displays calcium-dependent *exo*-PG activity [42]. Furthermore, *exo*-polygalacturonase activity had been observed during abscission events in citrus explants before [43]. *Endo*-PGs and *exo*-PGs seem to be both involved in cell elongation as well as cell separation events. Therefore, their enzymatic activity does not directly correlate with one or the other process but rather a combination of both activities seems to be necessary in both events. Hence, it is tempting to speculate if cell elongation and cell separation in principal rely on identical pectin modifications.

The Arabidopsis genome contains 69 PG genes [35,44]. Several of these arose from tandem duplications and belong to the same phylogenetic subclade [21]. Intuitively, one would speculate that these tandem-duplicated genes might act in a redundant manner if the expression pattern of these genes did not diverge. Only for a handful of PG genes is there direct evidence for their function during development, based on loss-of-function phenotypes. For many other Arabidopsis PG genes, the lack of an obvious loss-of-function phenotype might indeed be a result of genetic redundancy [18].

**Figure 1:**
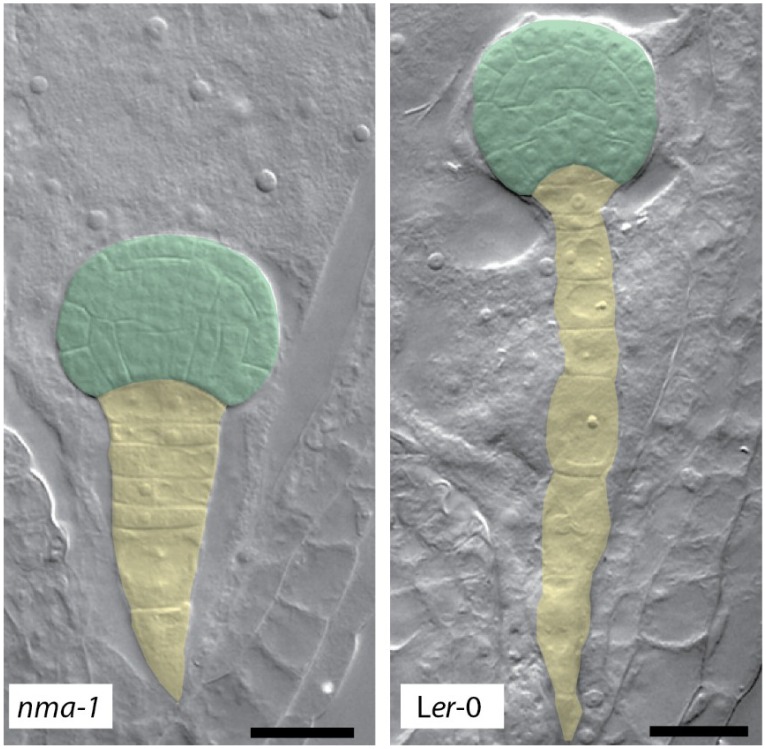
Embryonic phenotype of *nma* mutants. Suspensor cells in *nma-1*^−/−^ embryos show reduced cell elongation compared to wild-type. The embryo proper is false-colored in green, suspensor cells are false-colored in yellow. Scale bar: 20 µm.

Recent findings might shed some further light on this situation: Cell elongation defects were reported in embryos of the Arabidopsis *nimna* (*nma*) mutant [45]. *NMA* codes for a putative *exo*-polygalacturonase and is preferentially expressed in reproductive tissue. Cells of *nma* mutant embryos fail to elongate as early as the zygote stage and severe cell elongation defects can be further observed in the suspensor while cells of the embryo-proper seem to recover from their defects at later stages of embryo development (Figure 1) [45]. This might indicate that other polygalacturonases can take over NMA function in the embryo-proper.

**Figure 2:**
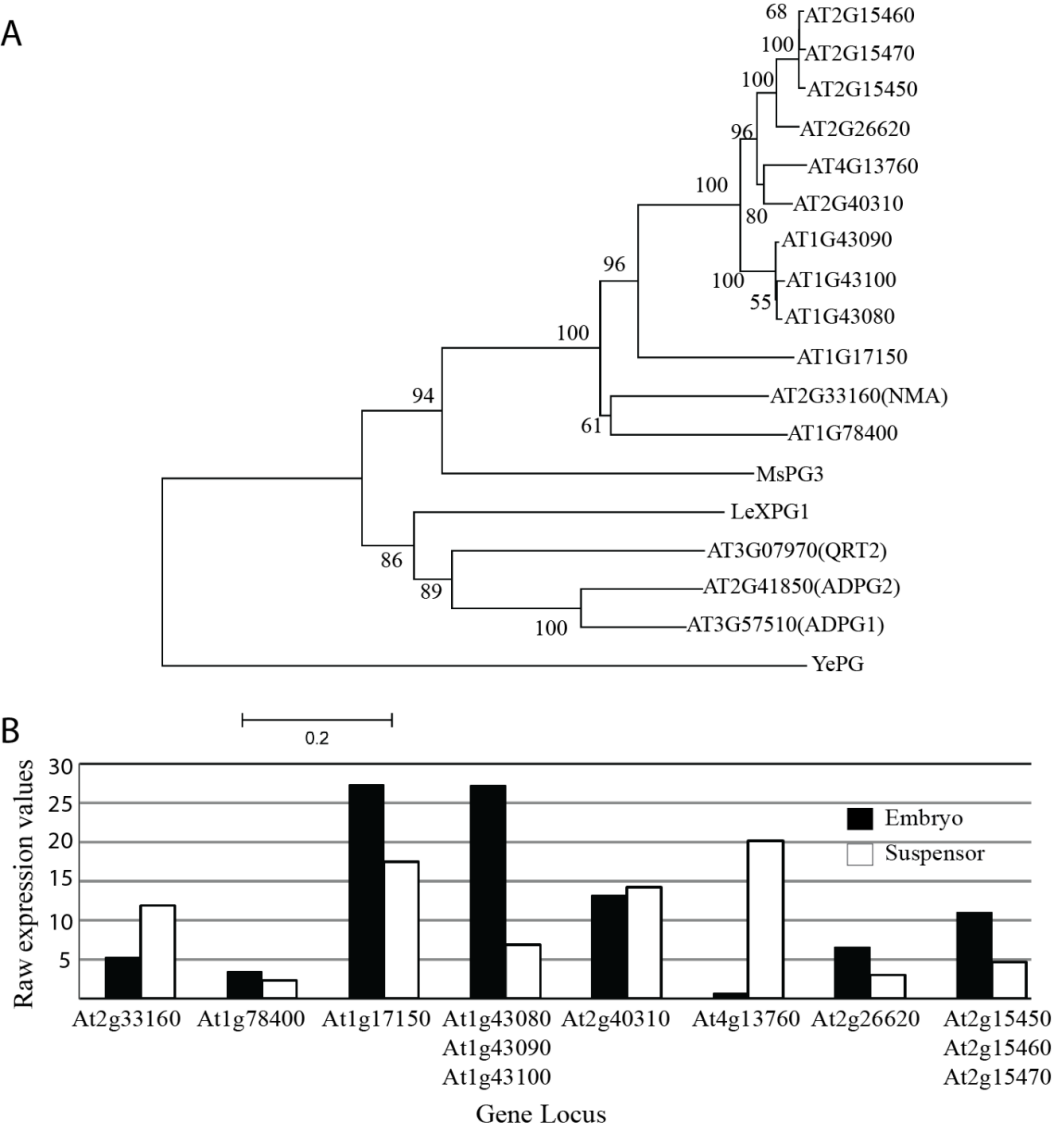
Phylogeny and expression values of *NMA*- and *QRT2*-related polygalacturonase genes. (**A**) Phylogenetic tree of NMA- and QRT2-related polygalacturonases. Phylogeny was created with protein sequences of two sub-clades A1a and A14 [21] using neighbour-joining with bootstrap values of 100 [49]. Some of the PGs mentioned in this review (*Medicago sativa* PG3, *Solanum lycopersicum* XPG1 and a bacterial *exo*-PG from *Yersinia enterocolitica*) were also included as outgroups. Scale bar represents amino acid substitutions per site; (**B**) Mean expression values of selected Arabidopsis polygalacturonase genes closely related to *NMA* in the embryo and suspensor based on publicly available microarray data [47].

Peptides from five PGs including NMA were found to be present in cell wall fractions of 5 day-old etiolated hypocotyls of *Arabidopsis* [46]. While *NMA* is obviously present in elongating hypocotyls, the *nma* mutation does not seem to have any measurable effect on hypocotyl length [45]. It appears that other PGs are able to compensate for the loss of NMA activity in this case. For the embryonic suspensor, the situation seems to be different: While there are several closely related PG genes expressed in the suspensor of globular stage embryos according to published microarray data (Figure 2) [47], the strong cell-elongation defects observed in *nma* mutants indicate that none of these can fulfill NMA function [45]. The reason for this might be different temporal expression, sub-cellular localization, enzyme activity, or substrate specificity.

A similar situation was observed for the closely related *ADPG1*, *ADPG2*, and *QRT2* genes (Figure 2) [40]. Loss of all three genes causes an impaired pod shatter phenotype and compromises anther dehiscence. While the *pADPG1::ADPG1* transgene was able to fully complement the pod shatter defects of the triple mutant, *QRT2* and the closely related PG gene *At1g48100* failed to do so when expressed under the *ADPG1* promoter [40]. Again, this would argue for a distinct function of these proteins in the cell separation process possibly caused by different enzymatic activity or substrate preference.

Expression analysis of three closely related tomato PG genes (*TAPGl*, *TAPG2*, and *TAPG4*) indicates temporal regulation during leaf and flower abscission. The temporal expression pattern of these genes suggests that they might act consecutively to fulfill a stepwise modification of the pectin matrix [48].

## 5. Perspectives

Pectin plays a central role in determining the physicochemical properties of the primary cell wall. Modifications of the pectin matrix are thus elementary for cell elongation by determining the extensibility of the cell wall. The degree of methyl-esterification is one important aspect that seems to be tightly regulated, but recent data emphasizes the importance of pectin-hydrolyzing polygalacturonases in cell elongation processes [41,45]. The temporally regulated activity of *endo-* as well as *exo*-PGs seems essential for both cell elongation and cell separation processes. To what degree the vast number of PG genes in the Arabidopsis genome reflects genetic redundancy or displays the need for a high number of specific enzymes in these cell wall processes is still unclear. With recently described Arabidopsis mutants like *nma* or *pgx1*, which show obvious and quantifiable loss-of-function phenotypes, this question can now be addressed. Promoter-swap and complementation experiments are powerful tools to support the biochemical analysis of these proteins. Complementation experiments with *endo*- and *exo*-PGs will allow a better understanding to what degree these enzymes are functionally redundant or are involved in separate non-exchangeable steps in pectin modification during cell elongation processes.

The active site in PG proteins is well conserved but substrate recognition motifs are not well understood [23]. *In vivo* complementation assays along with biochemical studies guided by protein-structure data might help unraveling specific modes of substrate recognition.

Technical advances have greatly helped our understanding of the primary cell wall composition [5,50,51,52]. Studying the effect of well-characterized PGs might indirectly give further insight in cell wall composition and the structure and modifications of the pectin matrix.

Furthermore, with the recent advances in genome-editing tools, like the CRISPR/Cas9 system and their application in plant biology, the study of many tandem-duplicated PG genes is now technically possible [53,54].

Understanding the substrate-specificity and the nature of the pectin modification carried out by specific polygalacturonases will not only increase our understanding of plant cell wall biology during cell elongation but will also be valuable for their use in commercial products and technical processes like biofuel production.
